# Combined detection of circulating tumor cells, α-fetoprotein heterogene-3 and α-fetoprotein in the early diagnosis of HCC for the prediction of efficacy, prognosis, recurrence after microwave ablation

**DOI:** 10.1186/s13027-021-00367-2

**Published:** 2021-05-10

**Authors:** Jian Zhou, Yue Zhu, Yi Li, Kun Liu, Fei He, Sihuan Xu, Xin Li, Li Li, Junfang Hu, Yan Liu

**Affiliations:** 1 Department of Infectious Diseases, Puren Hospital Affiliated to Wuhan University of Science and Technology, 430081 Wuhan, China; 2 Biological Cell Therapy Research Center, Puren Hospital Affiliated to Wuhan University of Science and Technology, 430081 Wuhan, China; 3 Department of Ultrasound Interventional Therapy, Tianyou Hospital Affiliated to Wuhan University of Science and Technology, 430064 Wuhan, China; 4 The Ministry of Science and Education, Puren Hospital Affiliated to Wuhan University of Science and Technology, 430081 Wuhan, China; 5 Department of Pharmacy, Puren Hospital Affiliated to Wuhan University of Science and Technology, 430081 Wuhan, China

**Keywords:** Circulating tumor cells, α-fetoprotein alloplasm 3, α-fetoprotein, Hepatocellular carcinoma, Microwave ablation

## Abstract

**Background:**

Early diagnosis can significantly improve treatment outcomes for hepatocellular carcinoma (HCC) patients. Currently, the dosage of serum alpha fetoprotein (AFP) is widely used in the diagnosis of HCC, but this biomarker has low specificity and may cause false positive or false negative results. Thus, it’s necessary to find and validate other serum tumor markers that in association for AFP would increase the sensitivity and the specificity in the HCC diagnosis. This study investigated the predictive value of combined of AFP, AFP-L3, and Circulating tumor cells (CTCs).

**Methods:**

A total of 105 patients with HCC after microwave ablation (MWA) were divided into non recurrence group, recurrence group, good prognosis (CR + PR group, CR: Complete remission, PR: Partial remission) and poor prognosis (SD + PD group, SD: Stable, PD: Progression). ROC curve was used to analyze the short-term efficacy, prognosis and clinical value of combined detection of the three indicators in predicting postoperative recurrence of HCC patients with MWA.

**Results:**

The positive rate of serum CTCs, AFP-L3 and AFP combined detection in the diagnosis of HCC is higher than that of single index and two index detection. The AUC, sensitivity and specificity of serum CTCs, AFP-L3 and AFP combined detection was better than that of single index and two indexes in patients with HCC after MWA.

**Conclusions:**

Combined detection of AFP, AFP-L3, and CTCs can effectively make up for the shortcomings of the detection with single and pairwise indicators. It can’t only diagnose HCC in early, but also has a high clinical value of predicting the short-term efficacy, prognosis and recurrence of HCC patients after MWA treatment.

## Background

Hepatocellular carcinoma (HCC), the seventh most common malignant tumor and the second highest cancer-related cause of death worldwide, has become a major public health problem threatening human life and health [1]. The pathogenic mechanism of HCC is not clear, which might be related to viral hepatitis, environmental factors, liver cirrhosis, and chemical carcinogens such as aflatoxin [2, 3]. Due to a lack of typical symptoms in the early stage of HCC, most HCC patients are already in the middle and late stages when diagnosed, missing the best opportunity for surgeries [4]. At present, the diagnosis of HCC mainly relies on imaging, but due to the radioactivity of imaging examinations, patients cannot take frequent checks, which reduce the probability of an early diagnosis. Instead, serum tumor markers are easy to operate and are favored by both patients and doctors. Currently, detection of serum α-fetoprotein (AFP) level is the most used methods in the diagnosis of HCC. But, AFP does not have a strong specificity in the diagnosis of HCC, and there are certain limitations on the application [5]: the AFP level would increase when patients are in pregnancy or get embryonic cancer; moreover, AFP is in close link with the primary site, tumor type, and degree of cell differentiation, which might lead to the reports of false positives or false negatives. Thus, it has always been a hot spot on clinical research to explore more sensitive and specific serum tumor markers for the early diagnosis of HCC.

Current HCC treatment methods include surgical resections, liver transplantation, hepatic artery chemo embolization (TACE), radiotherapy and chemotherapy, radiofrequency ablation (RFA), microwave ablation (MWA), etc. of which MWA has been more and more widely used due to its safety, minimal invasiveness, simplicity and economy [6, 7]. MWA is a local treatment method that causes tumor cell coagulation and necrosis by generating heat through the rotation of dipole molecules. Although MWA gets many advantages in the treatment of HCC, there are also certain limits. Researchers found that there is still 6.6-8.5 % of tumor recurrence of the lesion after liver cancer ablation treatment; in fact, incomplete ablation often result in a much higher recurrence rate [8, 9]. It thus remains a clinical challenge to effectively predict the short-term curative effect, prognosis and postoperative recurrence of HCC patients after MWA treatment. At present, AFP quantitative detection combined with imaging examination has been a method most frequently used in clinical evaluation. However, neither the sensitivity nor the specificity of a single AFP detection is satisfactory enough, nor imaging can’t be taken as a frequently used detection method, it is necessary to find a new prediction method.

Since AFP is not sensitive and specific enough in early diagnosis of HCC, we need to rely on two or more tumor markers to improve the diagnostic efficiency [10]. The AFP heterogeneous body can be divided into 3 types, which are AFP-L1, AFP-L2 and AFP-L3. AFP-L1 comes from benign liver disease, and it is the main component of AFP, AFP-L2 mainly comes from pregnant women, and AFP-L3 is unique to liver cancer cells. The level of AFP-L3 in serum will increase linearly with the growth of hepatocellular carcinoma, which has a relatively high specificity for the diagnosis of liver cancer, and it will not be restricted by the rule of AFP ≥ 400 µg/L. If the patients’ serum AFP level increased slightly, but AFP-L3 > 15 %, it may indicate the existence of HCC [11]. Many reports pointed out that AFP-L3 could be used as a marker for HCC auxiliary examination, and named it a new generation of liver cancer markers [12, 13]. However, studies have found that the AFP-L3 level of 15-30 % of AFP-positive liver cancer patients is below 10 %, which means a low percentage of AFP-L3 cannot rule out the presence of liver cancer [14].

Circulating tumor cells (CTCs) refer to tumor cells that come from primary tumors or metastases invading the peripheral blood of cancer patients through vascular invasion [15]. CTCs in the blood of patients with malignant tumors include epithelial tumor cells, epithelial-mesenchymal transition (EMT) tumor cells and tumor stem cells [16, 17]. It is reported that through modern techniques of immunology and molecular analysis, CTCs can be detected in the peripheral blood of patients with a variety of solid tumors, and CTCs is also closely related with tumor micro metastasis [18]. Compared with traditional detection methods such as tissue biopsy, CTCs detection of peripheral blood has such advantages of being real-time, efficient, convenient, less traumatic and highly reproducible [19–21]. It can not only be used to diagnose primary liver cancer in the early stage, but also dynamically monitor the short-term curative effects, as well as assessing the prognosis [22]. At present, CTCs is playing an important role in predicting the recurrence and prognosis of many tumors, but researches on its applications in liver cancer are far less than those in other types of tumor [23–27].

With the rapid progress of biochemical detection technology, targeted detection of cancer markers have reduced the rate of missed diagnosis, and provides much valuable reference data for early cancer diagnosis. But the detection with a single indicator would not lead to the improvement of diagnosis rate, and the rate of missed diagnosis still remains high. Selection of cancer markers with specific characteristics is getting to be the basis for an accurate cancer diagnosis and the key to a successful clinical diagnosis research. At present, diagnosis of combined indexes has become an effective method for the diagnosis of primary liver cancer. There are few reports on the application of combined detection of CTCs, AFP-L3 and AFP in the early diagnosis of HCC, especially when it comes to the prediction of the short-term efficacy, prognosis and recurrence after MWA. Therefore, this study intends to observe the clinical value of CTCs, AFP-L3 combined with AFP in the early diagnosis of HCC, and in the prediction of the short-term efficacy, prognosis, and postoperative recurrence after MWA.

## Materials and methods

### Clinical data

A total of 143 patients with HCC hospitalized in Puren Hospital affiliated to Wuhan University of Science and Technology, and Tianyou Hospital affiliated to Wuhan University of Science and Technology in the period between June 2014 to March 2017 were included as the research subjects. Meanwhile, 102 patients with liver cirrhosis (including alcoholic liver cirrhosis, viral hepatitis cirrhosis), 127 patients with hepatitis (including viral hepatitis, fatty liver, autoimmune liver disease, and drug-induced liver injury), and 110 healthy outpatients hospitalized in the same period were included as cirrhosis group, hepatitis group and normal control group respectively. This research project had been approved by the ethics committee of both hospitals.

### Criteria for case inclusion and exclusion

The inclusion criteria of HCC patients were as follows: ① 18–75 years old; ② diagnosis of HCC by histopathological examination; ③ be in accordance with the Standards for Diagnosis and Treatment of Hepatocellular Carcinoma of Chinese Guidelines (2017 Edition); ④ received no surgery, radiotherapy, chemotherapy or other treatments; ⑤ be clear about the research plan and signed the voluntary participation consent form. Exclusion criteria: ① with severe heart, lung, kidney, and brain diseases or dysfunction; ② with extra hepatic metastases; ③ with severe coagulopathy or bleeding tendency; ④ with bleeding of the upper gastrointestinal tract caused by portal hypertension within 3 months; ⑤ with severe liver cirrhosis or moderate ascites. The inclusion criteria for the cirrhosis group, hepatitis group and the normal control group were as follows: ① 18–75 years old; ② patients in the cirrhosis group and the hepatitis group were diagnosed by both laboratory and imaging examinations; ③ be aware of the research plan and signed the voluntary participation consent form. Exclusion criteria: ① lack of laboratory, clinical and medical history data; ② with severe hemolysis, microbial contamination or jaundice; ③ serum samples did not meet the standards of serum collection or processing; ④ quit midway from the study.

### Surgical methods

A total of 105 patients in the HCC group were treated with MWA, in which the microwave therapy instrument (Kangyou KY2000) was used at a regulating frequency of 2450 MHz, the continuous working mode was selected, the working power range was 10-100 W, and a hard internal water-cooled antenna (developed by Nanjing Qinghai Microwave Research Institute) was used. Kept patients in the supine position, took 5mL of 5 % Lidocaine and diluted with the same volume of normal saline, then performed tumor ablation under ultrasound guidance. The scope included the tumor tissue and the surrounding normal liver tissue within 0.5-1 cm. The selection of needle area and maximum section were based on the tumor site, to keep away from large blood vessels, bile ducts, gallbladder and gastrointestinal tract. Inserted a hard internal water-cooled antenna into the center of the tumor, set the power at 80-100 W according to the size of the tumor, and set the working time for 3–15 min, then turned on the microwave therapy instrument to perform MWA treatment. In the treatment of tumor whose diameter ≥ 2.5 cm, two antennas were used for punctures, and the distance between the two microwave needles was about 1.8 cm. During this process, patients’ arterial blood pressure, ECG, respiration and blood oxygen saturation were closely monitored. When the target area under ultrasound was completely covered by hyper echo, the microwave needle could be removed. Re-examination with enhanced CT was carried out within 48 h after surgery, if the tumor site was found to be incomplete necrotic, MWA treatment or anhydrous alcohol injection should be given immediately.

### Research methods

#### Detection of liver function and blood indicators

Test kits detecting indexes of serum liver function (ALT, AST, GGT, TBIL, DBIL, ALB) and blood routine index (Plt, WBC, RBC, Hb) were purchased from Beijing Zhongshan Biotech Technology Co., Ltd.; and analyzed with the Roche Cobas 6000 automatic biochemical immunoassay analyzer.

#### Detection of AFP-L3

Detection kits were provided by Beijing Rejing Biotechnology Co., Ltd.; micro spin column method was used for AFP-L3 separation, and analyzed with Roche Cobas 601 automatic biochemical immunoassay analyzer.

#### Detection of serum AFP

Cobas 6000 automatic biochemical immunoassay analyzer from Roche, Switzerland was used, with the original supporting reagent. The detection was carried out in strict accordance with the instructions of instruments and reagents, and the high and low values of quality control were all within the required range.

#### Detection of CTCs

Peripheral blood of 7.5 mL was drawn, and the CTCs detection system of Cell Seareh TM (Johnson & Johnson) was used to detect the number of CTCs in the blood. The detection system mainly included: Cell Save storage tube, Cell Search kit, Cell Tracks automatic processing system, magnetic tank, Cell Tracks automatic analyzer. After centrifugation, the blood samples stored in the Cell Save storage tube were subjected to a series of immune reactions in the Cell Tracks automatic processing system. Then incubated in a magnetic slot (the captured CTCs were moved to the analysis surface of the sample box with the action of a magnetic field to form a single cell layer). Finally, the Cell Track automatic analyzer scanned and analyzed the results, and the operator performed the final interpretation.

The positive threshold of each tumor marker was in accordance with the standard established by the Liver Cancer Committee of Chinese Anti-Cancer Association in 2001, which was, AFP ≥ 400 µg/L as positive; AFP-L3 ≥ 15 % as positive; CTCs ≥ 1 as positive.

### Short‐term efficacy

The evaluation criteria were as follows: (1) Complete remission (CR): All lesions disappeared after treatment, and all pathological lymph nodes had a short diameter of < 10 mm; (2) Partial remission (PR): After treatment, the total length of all lesions decreased for more than 30 %; (3) Stable (SD): After treatment, the total length of all lesions was reduced but not to the level of PR, or the total length of the lesions was increased but not reaching the level of PD; (4) Progression (PD): The total length of all target lesions increased by 20 % or more, or new lesions appeared. CR + PR was regarded as a good prognosis, and SD + PD was regarded as a poor prognosis.

### Follow‐up

Observation started the day when patients got discharged from the hospital, all patients were followed up by telephone, electronic communication and other means, together with patient return visit medical records. If a patient died halfway, the follow-up would be terminated, the lost patients would be excluded, and the overall survival (OS) of the patients would be counted. If a new lesion within 2 cm was found in or around the original ablation lesion within 6 months after the surgery, it would be diagnosed as a postoperative recurrence.

### Statistical analysis

SPSS 20.0 was used for data analysis, the measurement data of serum AFP-L3, AFP, CTCs levels were expressed as mean ± standard deviation, t-test was used for between-group comparison; F-test was used for comparison between multiple groups, SNK-q test was used for pairwise comparison; Chi-square test was used to compare the positive rate of AFP-L3 % between the two groups; ROC curve was used to analyze the clinical predictive value of single and combined detection of AFP-L3, AFP, CTCs, and *P* < 0.05 was considered as statistically different.

## Results

### Comparison of clinical data

Other than gender differences, the differences between groups were significant at all other indicators (*P* < 0.05). The patients in the HCC group were significantly older than patients in the cirrhosis group, hepatitis group and the normal control group (*P* < 0.05). The liver function indexes of the HCC group such as AST, GGT, TBIL, DBIL, ALB were significantly higher than those in cirrhosis group, hepatitis group and normal control group (*P* < 0.05). But the indexes of blood routines of the HCC group such as Plt, WBC, RBC, Hb were significantly lower than those in the cirrhosis group, hepatitis group and the normal control group, and the difference was statistically significant (*P* < 0.05), as shown in Table 1.

**Table 1:** Comparison of Clinical Data

Indexes	Normal control group (*n* = 110)	Hepatitis group (*n* = 127)	Cirrhosis group (*n* = 102)	HCC group (*n* = 143)	*P*
Age (years)	49.4(41.2-60.7)	48.6(37.9-61.2)	51.3(42.2-63.5)	57.8(46.3-67.6)	<0.001
Sex (n, %)					
Male	63(57.27)	70(55.12)	59(57.84)	91(63.64)	>0.05
Female	47(42.73)	57(44.88)	43(42.16)	52(36.36)	
ALT (U/L)	19.6(13.2-26.8)	30.2(21.6-50.9)	43.7(30.7-64.2)	70.3(40.1-115.4)	<0.001
AST (U/L)	16.3(10.1-23.5)	27.1(19.5-46.3)	40.2(26.0-59.1)	55.1(36.8-90.6)	<0.001
GGT (U/L)	17.1(12.5-30.1)	24.4(17.9-31.4)	36.0(27.2-43.5)	98.6(41.2-167.8)	<0.001
TBIL (μmol/L)	12.6(9.3-16.7)	18.2(13.0-25.6)	25.3(18.1-33.2)	35.1(20.9-58.3)	<0.001
DBIL (μmol/L)	5.1(3.7-7.3)	7.0(5.1-11.2)	10.4(7.2-12.8)	15.7(12.1-27.7)	<0.001
ALB (g/L)	43.7(41.2-46.1)	43.3(38.9-45.6)	37.6(33.7-39.6)	32.4(28.7-37.5)	<0.001
Plt (10^9^/L)	214.2(180.3-242.7)	196.5(174.2-219.5)	158.3(127.5-177.0)	129.7(93.8-151.6)	<0.001
WBC (10^9^/L)	5.7(5.0-6.4)	5.5(4.9-6.2)	4.3(3.1-5.2)	4.1(2.9-5.1)	<0.001
RBC (10^12^/L)	4.8(4.3-5.1)	4.7(4.2-5.2)	3.8(2.9-4.2)	3.2(2.5-3.8)	<0.001
Hb (g/L)	145.9(134.8-153.9)	141.7(130.2-150.6)	112.6(96.7-125.1)	90.7(72.7-115.1)	<0.001

*Abbreviations:ALB* albumin, *ALT* alanine aminotransferase, *AST* aspartate aminotransferase, *DBIL* direct bilirubin, *GGT* gamma-glutamyl transferase, *Hb* hemoglobin, *Plt* Platelets, *RBC* red blood cells, *TBIL* total direct and indirect bilirubin, *WBC* white blood cells

### Comparison of serum AFP, AFP-L3 and CTCs levels

The levels of serum AFP, AFP-L3, and CTCs of the HCC group were significantly higher than those in the cirrhosis group, hepatitis group and the normal control group (*P* < 0.05), as shown in Table 2.

**Table 2:** Comparison of Serum AFP, AFP-L3 and CTCs Levels

Groups	n	AFP(ng/mL)	AFP-L3(%)	CTCs(/mL)
Normal control group	110	7.16(5.27~10.05)	5.32(3.63-7.07)	0
Hepatitis group	127	9.37(6.54~12.86)	6.45(4.51-8.15)	0.37(0-0.52)
Cirrhosis group	102	22.08(13.02~178.91)^a,b^	12.73(9.07-15.32)^a,b^	1.53(0.42-2.15)^a,b^
HCC group	143	183.27(67.93~1512.34)^a,b,c^	17.07(13.32-21.91)^a,b,c^	2.47(1.95-2.93)^a,b,c^
*F*		112.02	67.16	78.37
*P*		<0.001	<0.001	<0.001

Note: vs. normal control group, ^a^*P*<0.05; vs. hepatitis group, ^b^*P*<0.05; vs. the other groups, ^c^*P*<0.05

### Comparison of positive rates of serum AFP, AFP-L3 and CTCs

The positive rates of single detection of serum AFP, AFP-L3, and CTCs indicated that CTCs detection had the highest positive rate in the HCC group (81.8 %). The positive rates of combined detection of serum AFP, AFP-L3, and CTCs were also compared, and it was found that in the HCC group, the positive detection rate of serum AFP paired with AFP-L3, serum AFP paired with CTCs, serum AFP-L3 paired with CTCs, serum AFP combined with AFP-L3 and CTCs increased to 88.1 %, 90.9 %, 93.0 and 97.2 %, respectively, as shown in Table 3.

**Table 3:** Comparison of Positive Rates of Serum AFP, AFP-L3 and CTCs [cases (%)]

Indexes	Normal control group(n=110)	Hepatitis group(n=127)	Cirrhosis group(n=102)	HCC group(n=143)	*χ*^2^	*P*
AFP	0(0.0)	5(3.9)	21(20.6)	92(64.3)	152.7	<0.001
AFP-L3	0(0.0)	4(3.1)	23(22.5)	105(73.4)	176.1	<0.001
CTCs	0(0.0)	2(1.6)	28(27.5)	117(81.8)	205.8	<0.001
AFP and AFP-L3	0(0.0)	7(5.5)	30(29.4)	126(88.1)	220.9	<0.001
AFP and CTCs	0(0.0)	6(4.7)	34(33.3)	130(90.9)	193.6	<0.001
AFP-L3 and CTCs	0(0.0)	5(3.9)	32(31.4)	133(93.0)	187.3	<0.001
AFP and AFP-L3 and CTCs	0(0.0)	8(6.3)	35(34.3)	139(97.2)	195.2	<0.001

### Analysis of the value of single and combined detection of serum AFP, AFP-L3, CTCs in the early diagnosis of HCC

**Fig. 1:**
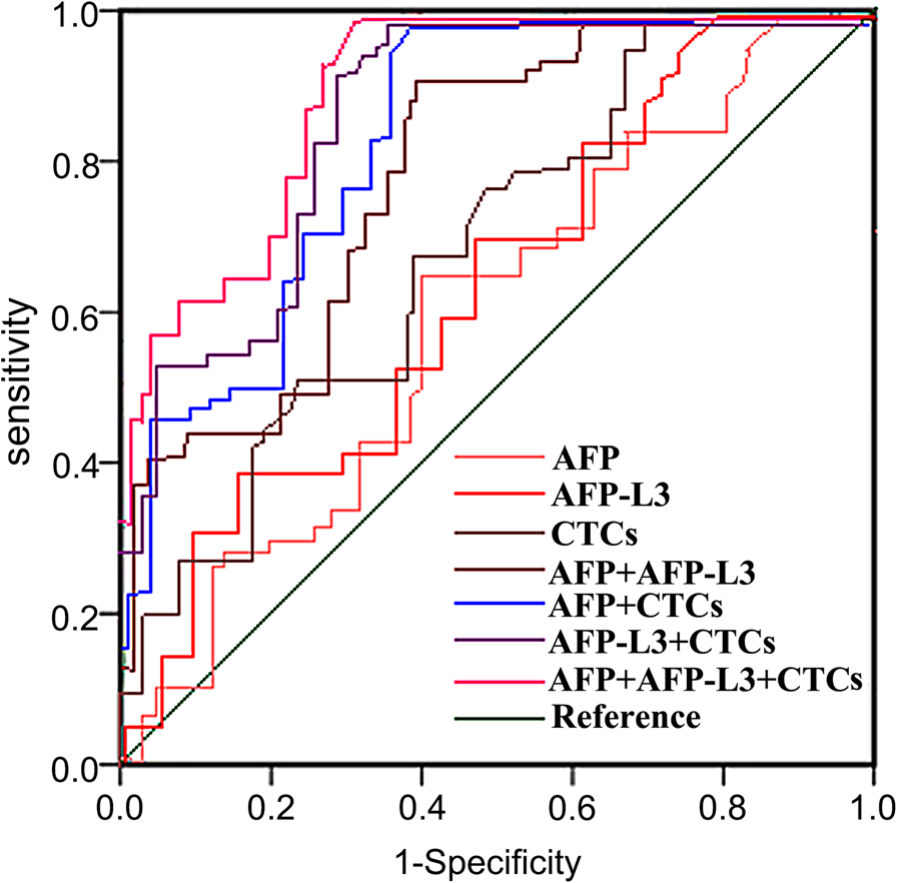
ROC Curve of Single and Combined Detection of Serum AFP, AFP-L3, CTCs in the Early Diagnosis of HCC

ROC curve was used to analyze the clinical value of single and combined detection of serum AFP, AFP-L3, and CTCs in the early diagnosis of HCC. Results showed that the combined detection of AFP, AFP-L3 and CTCs could improve the clinical value of early diagnosis of HCC, as shown in Table 4; Fig. 1.

**Table 4:** Analysis of the Value of Single and Combined Detection of Serum AFP, AFP-L3, CTCs in the Early Diagnosis of HCC

Index	AUC	*P*	Sensitivity	Specificity	Youden index
AFP	0.638	<0.001	0.643(92)	0.794(81)	0.437
AFP-L3	0.674	<0.001	0.734(105)	0.775(79)	0.509
CTCs	0.705	<0.001	0.818(117)	0.725(74)	0.543
AFP+AFP-L3	0.767	<0.001	0.881(126)	0.706(72)	0.587
AFP+CTCs	0.793	<0.001	0.909(130)	0.667(68)	0.576
AFP-L3+CTCs	0.817	<0.001	0.930(133)	0.686(70)	0.616
AFP+AFP-L3+CTCs	0.832	<0.001	0.972(139)	0.657(67)	0.629

### Comparison of serum AFP, AFP-L3 and CTCs levels between non-recurrent group and recurrent group relapsed before and after microwave ablation of liver cancer

The levels of serum AFP, AFP-L3 and CTCs in the recurrent group that relapsed before microwave ablation of liver cancer were higher than those in the non-recurrent group, and the difference was statistically significant (*P* < 0.05). The levels of serum AFP, AFP-L3 and CTCs in the non-recurrent group after microwave ablation of liver cancer were significantly higher than the levels before surgery (*P* < 0.05). The level of serum AFP, AFP-L3, and CTCs in the recurrent group after microwave ablation of liver cancer were significantly higher than the levels before surgery, and significantly higher than the levels detected in the non-recurrent group after surgery (*P* < 0.05). As shown in Table 5.

**Table 5:** Comparison of Serum AFP, AFP-L3, and CTCs Levels between Non-recurrent Group and Recurrent Group Relapsed before and after Microwave Ablation of Liver Cancer

Group	Time	Cases	AFP(ng/mL)	AFP-L3(%)	CTCs
Non-recurrence Group	Before surgery	57	157.34(67.93~508.71)	14.72(13.87~15.65)	2.23(1.95~2.53)
	6 months after surgery	57	62.17(38.57~107.76)^a^	7.01(5.13~8.93)^a^	1.70(1.52~1.91)^a^
Recurrence Group	Before surgery	48	216.82(97.12~731.85)^a^	17.51(15.44~19.26)^a^	2.65(2.14~2.93)^a^
	6 months after surgery	48	351.06(216.84~485.29)^ab^	21.38(18.91~23.73)^ab^	3.05(2.62~3.31)^ab^

Note: Compared with the non-recurring group before surgery, ^a^*P*<0.05; compared with the non-recurring group at 6 months after surgery, ^b^*P*<0.05

### The value of single and combined detection of serum AFP, AFP-L3, CTCs in predicting the recurrence of HCC patients after microwave ablation

**Fig. 2:**
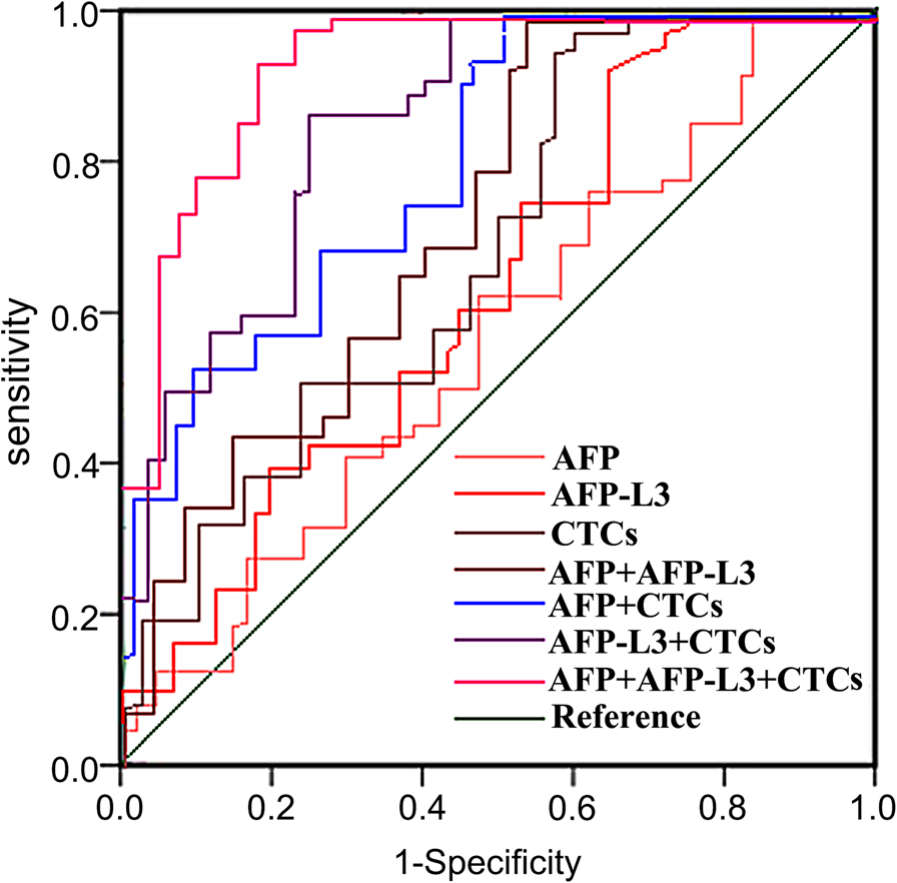
ROC Curve of Single and Combined Detection of Serum AFP, AFP-L3, CTCs in Predicting the Recurrence in HCC Patients after Microwave Ablation

ROC curve was used to analyze the clinical value of single and combined detection of serum AFP, AFP-L3, CTCs in predicting the recurrence of HCC patients after microwave ablation. The results showed that a combined detection of serum AFP, AFP-L3, CTCs could improve the prediction of recurrence in HCC patients after microwave ablation. As shown in Table 6; Fig. 2.

**Table 6:** The Value of Single and Combined Detection of Serum AFP, AFP-L3, CTCs in Predicting the Recurrence of HCC Patients after Microwave Ablation

Index	AUC	*P*	Sensitivity	Specificity	Youden index
AFP	0.552	<0.001	0.667(32)	0.614(35)	0.281
AFP-L3	0.619	<0.001	0.729(35)	0.684(39)	0.413
CTCs	0.651	<0.001	0.750(36)	0.702(40)	0.452
AFP+AFP-L3	0.703	<0.001	0.792(38)	0.737(42)	0.529
AFP+CTCs	0.767	<0.001	0.833(40)	0.754(43)	0.587
AFP-L3+CTCs	0.841	<0.001	0.854(41)	0.789(45)	0.643
AFP+AFP-L3+CTCs	0.917	<0.001	0.896(43)	0.825(47)	0.721

### Analysis of the relationship between serum AFP, AFP-L3, CTCs levels and the short-term efficacy of microwave ablation in HCC patients

The preoperative levels of serum AFP, AFP-L3, and CTCs in SD + PD group were higher than the levels in the CR + PR group, and the difference was statistically significant (*P* < 0.05). In CR + PR group, the levels of serum AFP, AFP-L3 were decreased compared with the levels before surgery, and the difference was statistically significant (*P* < 0.05). The levels of serum AFP, AFP-L3, and CTCs in SD + PD group 3 months after surgery were higher than the preoperative levels, and higher than the postoperative levels in the CR + PR group, the differences were statistically significant (*P* < 0.05), as shown in Table 7.

**Table 7:** Analysis of the Relationship between Serum AFP, AFP-L3, CTCs Levels and Short-term Efficacy after Microwave Ablation in HCC Patients

Group	Time	Cases	AFP(ng/mL)	AFP-L3(%)	CTCs
CR+PR	Before surgery	69	172.44(67.93~612.09)	15.36(13.87~16.37)	2.30(1.95~2.61)
	3 months after surgery	69	54.38(30.25~89.51)^a^	6.68(4.92~8.15)^a^	1.63(1.49~1.88)^a^
SD+PD	Before surgery	36	264.51(117.87~731.85)^a^	18.53(16.20~19.26)^a^	2.64(2.22~2.93)^a^
	3 months after surgery	36	326.82(193.20~437.36)^ab^	20.91(18.65~23.51)^ab^	2.95(2.54~3.21)^ab^

Note: Compared with CR + PR group before surgery, ^a^*P*<0.05; compared with CR + PR group 3 months after surgery, ^b^*P*<0.05

### The value of single and combined detection of serum AFP, AFP-L3, CTCs in predicting the short-term curative effect of HCC patients after microwave ablation

**Fig. 3:**
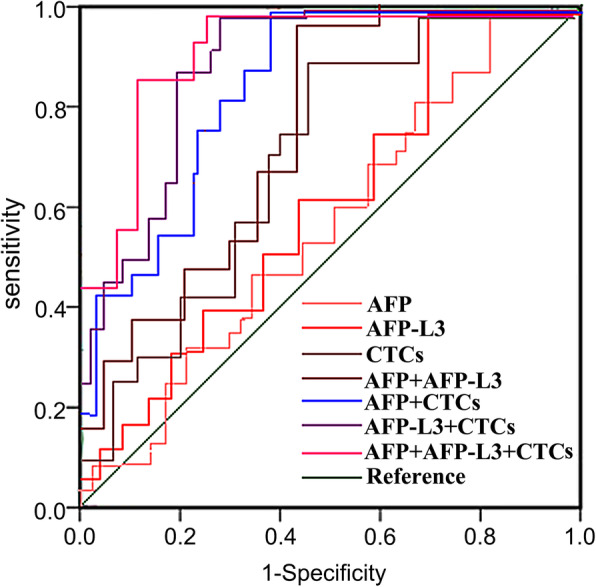
TROC Curve of Single and Combined Detection of Serum AFP, AFP-L3, CTCs in Predicting the Poor Short-term Efficacy of HCC Patients after Microwave Ablation

ROC curve was used to analyze the clinical value of single and combined detection of serum AFP, AFP-L3, CTCs in predicting the short-term efficacy of microwave ablation in HCC patients. Results showed that the combined detection of serum AFP, AFP-L3, and CTCs could improve the prediction of poor short-term efficacy of HCC, as shown in Table 8; Fig. 3.

**Table 8:** The Value of Single and Combined Detection of Serum AFP, AFP-L3, CTCs in Predicting the Poor Short-term Efficacy after Microwave Ablation in HCC Patients

Index	AUC	*P*	Sensitivity	Specificity	Youden index
AFP	0.532	<0.001	0.667(24)	0.594(41)	0.261
AFP-L3	0.586	<0.001	0.722(26)	0.652(45)	0.374
CTCs	0.659	<0.001	0.778(28)	0.681(47)	0.459
AFP+AFP-L3	0.746	<0.001	0.861(31)	0.696(48)	0.557
AFP+CTCs	0.781	<0.001	0.889(32)	0.710(49)	0.599
AFP-L3+CTCs	0.837	<0.001	0.917(33)	0.725(50)	0.642
AFP+AFP-L3+CTCs	0.896	<0.001	0.944(34)	0.768(53)	0.712

### Analysis of the relationship between serum AFP, AFP-L3, CTCs levels and the prognosis of microwave ablation in HCC patients

**Fig. 4:**
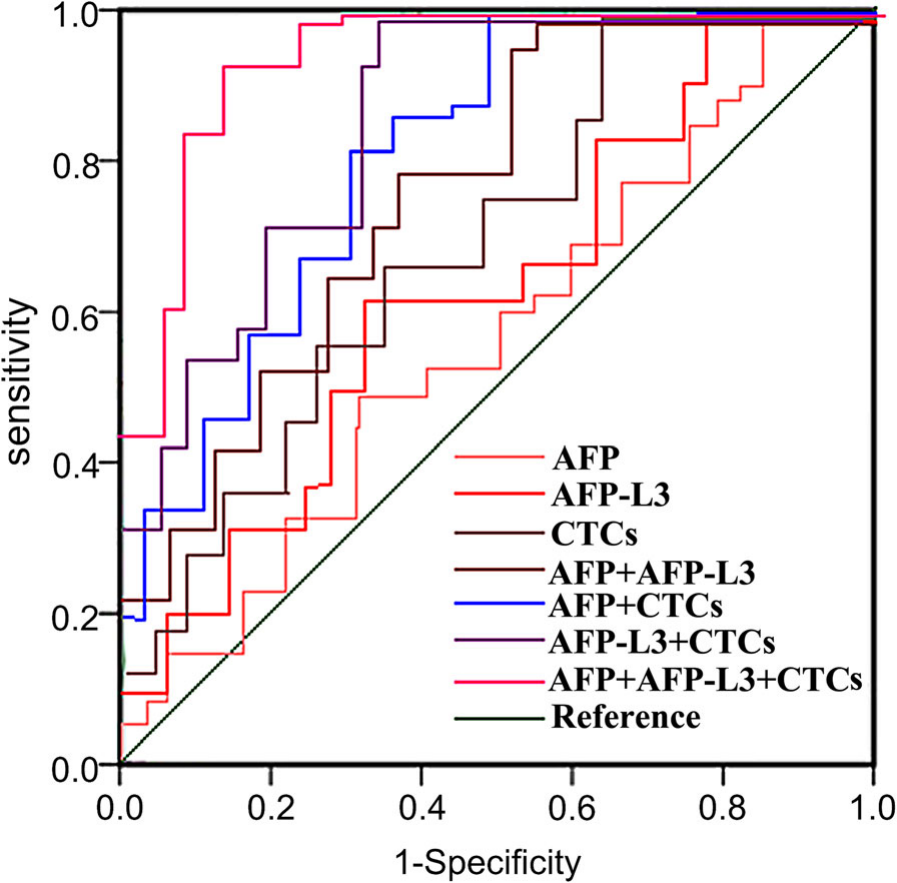
ROC Curve of Single and Combined Detection of Serum AFP, AFP-L3, CTCs in Predicting the Level of OS < 3 years after Microwave Ablation in HCC Patients

The patients were followed up for 3 years, 13 patients that lost during the follow-up period were excluded, and the patients were divided into OS > 3 years group and OS < 3 years group. The results showed that the serum AFP, AFP-L3 and CTCs levels of patients in the OS > 3 years group were lower than those in the OS < 3 years group, and the differences were statistically significant (P < 0.05), see Table 9; Fig. 4.

**Table 9:** Analysis of the Relationship between Serum AFP, AFP-L3, CTCs Levels and Prognosis of HCC Patients Treated with Microwave Ablation

Group	Case	AFP(ng/mL)	AFP-L3(%)	CTCs
OS>3 years	40	162.28(67.93~427.05)	14.95(13.87~15.84)	2.25(1.95~2.53)
OS<3 years	52	270.33(154.62~731.85)^a^	18.81(16.32~19.26)^a^	2.72(2.33~2.93)^a^

Note: Compared with the group of OS > 3 years, ^a^*P*<0.05

### The value of single and combined detection of serum AFP, AFP-L3, CTCs in predicting the level of OS < 3 years of HCC patients after microwave ablation

ROC curve was used to analyze the clinical value of single and combined detection of serum AFP, AFP-L3, CTCs in predicting the prognosis of HCC patients treated with microwave ablation. Results showed that the combined detection of AFP, AFP-L3, CTCs could improve the prediction of the levels of OS < 3 years for HCC patients treated with microwave ablation. As shown in Table 10.

**Table 10:** The Value of Single and Combined Detection of Serum AFP, AFP-L3, CTCs in Predicting the Level of OS < 3 years for HCC Patients after Microwave Ablation

Index	AUC	*P*	Sensitivity	Specificity	Youden index
AFP	0.546	<0.001	0.673(35)	0.625(25)	0.298
AFP-L3	0.595	<0.001	0.712(37)	0.675(27)	0.387
CTCs	0.647	<0.001	0.750(39)	0.700(28)	0.450
AFP+AFP-L3	0.749	<0.001	0.808(42)	0.750(30)	0.558
AFP+CTCs	0.820	<0.001	0.846(44)	0.775(31)	0.621
AFP-L3+CTCs	0.857	<0.001	0.865(45)	0.800(32)	0.665
AFP+AFP-L3+CTCs	0.923	<0.001	0.904(47)	0.825(33)	0.729

## Discussion

HCC is a highly malignant tumor, a majority of HCC patients are already in the late stage when diagnosed, thus leading to a poor prognosis. Early diagnosis and treatment are effective methods to prolong the survival time of HCC patients [28]. Due to the radiological nature of imaging examinations, patients cannot be routinely examined. Therefore, more and more research turn to focus on the discovery of HCC through serum tumor markers. At present, AFP is a serum tumor marker often used to monitor HCC, and it locates on the ribosomes of the rough endoplasmic reticulum of liver cells. When HCC occurs, the metabolism of patients gets accelerated, which in turn promotes the synthesis of AFP. Therefore, a significant increase of serum AFP levels can indicate the occurrence of HCC. However, serum AFP in some HCC patients are negative or in a low level, and the level of serum AFP also gets increased in some patients with benign liver diseases, which leads to a significant increase of misdiagnosis rate of HCC [29]. Therefore, it is necessary to combine serum AFP detection with other serological indicators to achieve an early diagnosis of HCC.

This study explored the clinical value of three markers of AFP, AFP-L3 and CTCs in the diagnosis of HCC, liver cirrhosis, and hepatitis, when used alone or in combination. Firstly, the expression levels of serum AFP, AFP-L3 and CTCs in different groups were compared, and the results showed that the levels of serum AFP, AFP-L3 and CTCs of patients in the HCC group were significantly higher than those in other groups. A comparison of the positive rates of serum AFP, AFP-L3, and CTCs showed that the positive rate of CTCs in the HCC group was the highest. Besides, a comparison of the positive rate of combined detection of AFP, AFP-L3 and CTCs indicated that the positive rates in the HCC group increased significantly when AFP was paired with AFP-L3, or with CTCs, or combined with both AFP-L3 and CTCs, or when AFP-L3 was paired with CTCs. The data above indicated that when AFP is combined with CTCs and AFP-L3 in the detection, it would play a complementary role in the clinical diagnosis of HCC. The rational use of these markers can promote the positive rate and the accuracy of diagnosis; meanwhile, it helps to reduce the rate of missed diagnosis and misdiagnosis.

ROC curve was used to analyze the clinical value of single and combined detection of serum AFP, AFP-L3, and CTCs in the early diagnosis of HCC. The results showed that a combined detection of AFP, AFP-L3 and CTCs increased the early diagnosis of HCC, and it’s better than the detection of single indicator or paired indicators. AFP-L3 is a specific α-fetoprotein generated by liver cancer tissues, and it is mainly found in the serum of HCC patients. The increase of patients’ serum level may be linked to the increasing number of AFP-L3 molecules generated by liver cancer cells, which reflects the heterogeneity of benign and malignant cells [30]. The increase of AFP-L3 relies not on the increase of AFP, but is only linked with the benignity and malignancy of liver diseases. Therefore, when the serum AFP level of HCC patients is not highly expressed, AFP-L3 would increase significantly, which means it can play a crucial role in HCC early diagnosis [31]. Compared with traditional detection methods such as tissue biopsy, CTCs detection is real-time, highly efficient and reproducible [19]. The CTCs detection has become a promising form of detection and drug target in the researches on early diagnosis, prognostic evaluation and monitoring of HCC, recurrence and metastasis mechanisms. In addition, CTCs detection could develop a multi-marker model when combined with other biomarker detection methods, promoting the sensitivity and specificity of diagnosis to a level above 90 % [32], which is consistent with the results of this study.

In recent years, with the development of minimally invasive technology and the renewal of treatment concepts, surgical resection is no longer the only choice in the treatment of HCC. MWA is a new technique of local thermal ablation, which featured as the rapid rise of temperature, easy to operate and repeatable. It can evaporate water near the lesion quickly through the high-frequency vibration heating mechanism of dipole and ion, leading to the degeneration, coagulation, and necrosis of tumor cells. MWA also could speed up blood clotting, resulting in a complete necrosis in the coagulation area, and realize the local inactivation [33, 34]. However, MWA treatment of HCC has been found to have certain limitations in clinical practice. Since part of the tumor lesion cannot be completely ablated, it may lead to local recurrence after treatment. A new method is thus needed to effectively predict the short-term efficacy, prognosis and recurrence of HCC patients after MWA treatment. In this study, HCC patients treated with MWA were divided into non-recurrent group and recurrent group by considering whether new tumor lesions appeared within 6 months after treatment. The patients were then divided into CR + PR group (good prognosis) and SD + PD group (poor prognosis) according to the relevant standards of short-term efficacy, they were further divided into OS > 3 years group and OS < 3 years group based on the data of overall survival (OS). The results showed that the levels of serum AFP, AFP-L3, and CTCs of patients in non-recurrent group, CR + PR group, and OS > 3 years group were significantly lower than those of patients in recurrent group, SD + PD group and OS < 3 years group (*P* < 0.05), suggesting that serum AFP, AFP-L3, and CTCs may be valuable indicators in predicting the short-term efficacy, prognosis and postoperative recurrence of HCC patients after MWA treatment.

ROC curve was used to further analyze the clinical value of single and combined detection of serum AFP, AFP-L3, CTCs in predicting the short-term efficacy, prognosis and postoperative recurrence of HCC patients after MWA treatment. The results indicated that a combined detection of these indicators could significantly improve the levels of AUC, sensitivity and specificity, and it had a better predictive value than single or paired indicators. AFP-L3 is a new generation of liver cancer biomarker, and as a specific α-fetoprotein generated by liver cancer cells, it has a high accuracy, specificity, and sensitivity. AFP-L3 is often used in combination with AFP in the clinical diagnosis, efficacy judgment, prognosis evaluation, and postoperative recurrence monitoring of HCC patients [35]. It has been found in the study that the changes of CTCs in patients’ peripheral blood after treatment can reflect the treatment effect. The decrease or disappearance of CTCs in patients’ peripheral blood often indicates a good treatment effect, if CTCs in patients’ peripheral blood remain unchanged or increased again after initial decrease, it often indicates an unfavorable treatment effect and a tumor recurrence, or indicates a drug resistance. Ye et al. [36] studied the relationship between CTCs and the clinical outcome of patients of hepatitis B-related hepatocellular carcinoma who received radical resection, and found that the DFS and OS of patients with lower CTCs were significantly prolonged compared with patients with higher CTCs, suggesting that higher CTCs might be an independent signal of poor prognosis of HCC patients. Yu et al. [37] studied the changes of CTCs in HCC patients after hepatectomy, and the results indicated that patients with increased CTCs after surgery had significantly shorter DFS and OS compared with patients without CTCs increase; patients had the worst prognosis when CTCs ≥ 2 before and after the surgery, and patients would have the longest DFS and OS when CTCs < 2. Chen et al. [38] retrospectively analyzed the CTCs levels and histopathologic types of 195 patients with hepatocellular carcinoma, and concluded that the total number of CTCs was related to BCLC staging, metastasis, and serum AFP levels. They also found recurrent patients had higher levels of mixed CTCs and mesenchymal CTCs, indicating that the number of CTCs and EMT classification were related to the prognosis of liver cancer. The above research results show that HCC patients’ CTCs levels after surgery are significantly linked with the efficacy, prognosis, and postoperative recurrence. Therefore, the combined detection of serum AFP, AFP-L3 and CTCs has a good predictive value.

## Conclusions

In summary, the combined detection of serum AFP, AFP-L3, and CTCs can effectively make up for the shortcomings of detection of single and pairwise indicators. It can not only be used for early diagnosis of HCC, but also has a good clinical value in predicting the short-term efficacy, prognosis and recurrence of HCC patients after MWA. Such a detection method is simple, stable, reliable, economical and practical, and is suitable for application in hospitals at all levels.

## Data Availability

All data generated or analysed during this study are included in this published article.

## Abbreviations

HCC: Hepatocellular carcinoma
CTCs: Circulating Tumor Cells
AFP-L3: α-fetoprotein Heterogene-3
AFP: α-fetoprotein
MWA: Microwave ablation
TACE: Transplantation, hepatic artery chemo embolization
RFA: Radiofrequency ablation
EMT: Epithelial-mesenchymal transition
CR: Complete remission
PR: Partial remission
SD: Stable
PD: Progression
OS: Overall survival
